# A randomised multicentre trial of acupuncture in patients with seasonal allergic rhinitis – trial intervention including physician and treatment characteristics

**DOI:** 10.1186/1472-6882-14-128

**Published:** 2014-04-06

**Authors:** Miriam Ortiz, Claudia M Witt, Sylvia Binting, Cornelia Helmreich, Josef Hummelsberger, Florian Pfab, Michael Wullinger, Dominik Irnich, Klaus Linde, Bodo Niggemann, Stefan N Willich, Benno Brinkhaus

**Affiliations:** 1 Institute of Social Medicine, Epidemiology and Health Economics, Charité-Universitätsmedizin Berlin, Berlin, Germany; 2 Institute for Complementary and Integrative Medicine, University Hospital Zurich, Zurich, Germany; 3 International Society for Chinese Medicine, Munich, Germany; 4 Department of Dermatology and Allergy, Technische Universität München, Munich, Germany; 5 Department of Prevention and Sports Medicine, Technische Universität München, Munich, Germany; 6 Department of Anesthesiology, Technische Universität München, Munich, Germany; 7 Institute of General Practice, Klinikum rechts der Isar, Ludwig Maximilians University, München, Germany; 8 Department of Pediatrics, Division of Pneumonology and Immunology, Charité-Universitätsmedizin, Berlin, Germany

**Keywords:** Acupuncture, Randomised controlled trial, Seasonal allergic rhinitis, Trial intervention, Sham acupuncture

## Abstract

**Background:**

In a large randomised trial in patients with seasonal allergic rhinitis (SAR), acupuncture was superior compared to sham acupuncture and rescue medication. The aim of this paper is to describe the characteristics of the trial’s participating physicians and to describe the trial intervention in accordance with the STRICTA (Standards for Reporting Interventions in Controlled Trials of Acupuncture) guidelines, to make details of the trial intervention more transparent to researchers and physicians.

**Methods:**

ACUSAR (ACUpuncture in Seasonal Allergic Rhinitis) was a three-armed, randomised, controlled multicentre trial. 422 SAR patients were randomised to semi-standardised acupuncture plus rescue medication (RM, cetirizine), sham acupuncture plus RM or RM alone. We sent a questionnaire to trial physicians in order to evaluate their characteristics regarding their education about and experience in providing acupuncture. During the trial, acupuncturists were asked to diagnose all of their patients according to Chinese Medicine (CM) as a basis for the semi-standardised, individualized intervention in the acupuncture group. Every acupuncture point used in this trial had to be documented after each session

**Results:**

Acupuncture was administered in outpatient clinics by 46 (mean age 47 ± 10 years; 24 female/ 22 male) conventionally-trained medical doctors (67% with postgraduate specialization such as internal or family medicine) with additional extensive acupuncture training (median 500 hours (1st quartile 350, 3rd quartile 1000 hours with 73% presenting a B-diploma in acupuncture training (350 hours)) and experience (mean 14 years in practice). The most reported traditional CM diagnosis was ‘wind-cold invading the lung’ (37%) and ‘wind-heat invading the lung’ (37%), followed by ‘lung and spleen qi deficiency’ (9%). The total number of needles used was higher in the acupuncture group compared to the sham acupuncture group (15.7 ± 2.5 vs. 10.0 ± 1.6).

**Conclusions:**

The trial interventions were provided by well educated and experienced acupuncturists. The different number of needles in both intervention groups could be possibly a reason for the better clinical effect in SAR patients. For future trials it might be more appropriate to ensure that acupuncture and sham acupuncture groups should each be treated by a similar number of needles.

**Trial registration:**

ClinicalTrials.gov: NCT00610584.

## Background

Over the past decade an increasing number of randomised controlled trials have been conducted to determine the efficacy or effectiveness of acupuncture in patients with allergic rhinitis. As shown by previous systematic reviews of acupuncture for the treatment of allergic rhinitis, the evidence on the specific effects of acupuncture is still inconclusive [1,2]. The trials included in those reviews have suffered from a variety of methodological limitations, such as small patient numbers or the lack of a sham-acupuncture control group.

In the randomised multicentre ACUpuncture in Seasonal Allergic Rhinitis (ACUSAR) trial, we investigated whether a semi-standardised acupuncture intervention plus rescue medication (RM) was more effective than standardised sham acupuncture plus RM or RM alone in patients with seasonal allergic rhinitis (SAR). The ACUSAR was the first acupuncture trial funded by the German Research Foundation (Deutsche Forschungsgemeinschaft, DFG). The DFG had requested a randomised trial including a sham control and a hierarchical test procedure with a non-inferiority and superiority procedure primarily for the comparison of acupuncture with sham acupuncture. The protocol and primary results have been published elsewhere [3,4].

In 2001 the STRICTA (Standards for Reporting Interventions in Controlled Trials of Acupuncture) guidelines were published to encourage more precise descriptions of the interventions used in controlled trials of acupuncture in publications and to improve the quality of these interventions [5]. The aim of this paper is to describe the characteristics of the trial’s participating physicians, to describe the trial intervention in accordance with the STRICTA (Standards for Reporting Interventions in Controlled Trials of Acupuncture) guidelines and to make details of the trial intervention more transparent to researchers and physicians.

## Methods

The ACUSAR trial was a randomised, controlled multicentre trial comparing acupuncture (Acu) plus rescue medication (RM) with non-penetrating sham acupuncture (Sham) plus RM and with RM alone in the treatment of SAR. Main inclusion criteria were SAR diagnosed by an allergist; IgE positivity to grass and birch pollen; age 16 to 45 years; no contraindications to cetirizine as anti-allergy medication; and ability to complete a symptom diary, including recording RM use. Main exclusion criteria were perennial AR, allergic asthma, moderate to severe atopic dermatitis, autoimmune disorders, severe chronic inflammatory diseases, specific immunotherapy during the past 3 years or planned in the next 2 years, previous acupuncture treatment for SAR, and any CAM use.

Patients were centrally randomised (ratio 2:1:1) in blocks of 8 to one of the three treatment groups. The randomisation schedule was generated using DatInf RandList, version 1.2 (DatInf, Tübingen, Germany) at the University of Hamburg, Hamburg, Germany. An independent clinical trials unit (KKS Charite’) implemented the allocation schedule using a centralised telephone randomisation procedure. Patients, trial statisticians, outcome assessors, data entry personnel, and the funder were blinded to treatment assignment throughout the trial. This trial followed the Declaration of Helsinki Good Clinical Practice guidelines (ICH-GCP) for trial conduct and included an external audit. All trial participants provided written informed consent and were not reimbursed for participating in the trial. The trial protocol was approved by the appropriate ethical review boards (ethics commissions and reference numbers: Charité- Universitätsmedizin Berlin: EA1/214/07; Landesärztekammer Bavaria: 7/08030; Landesärztekammer Brandenburg: AS(18)a/2008; Technical University Dresden Faculty of Medicine Carl Gustav Carus: EK315122008; Essen University Hospital: 08–3610; Jena University Hospital: 2248-03/08; Medical faculty Ludwig-Maximilian-University München: 105–08).

### Trial physicians

The participating physicians were recruited in a manner designed to ensure that their qualifications were adequate to perform a treatment procedure which included a CM diagnosis before acupuncture. According to the trial protocol [3], trial physicians had to fulfil the following criteria: (1) acupuncture training of at least 140 hours (equals an ‘A-diploma’ from the major German acupuncture associations); (2) at least 3 years of practical experience with acupuncture; (3) 50% of participating physicians had to have at least 350 h of acupuncture training (equals ‘B-diploma’); (4) 50% of trial physicians had to have experience working in clinical studies; and (5) required participation in trial training sessions on trial methods, applied trial interventions, and standards for performing clinical trials (ICH-GCP). At the trial beginning of the trial we sent a questionnaire to all 46 trial physicians. This questionnaire included 14 items on their medical background and training such as medical specialisation, scope of training as acupuncturist, time working as acupuncture specialist, qualification as trainer for acupuncture, CM diagnostic procedures, etc.

### CM syndrome diagnosis and trial intervention

To find a consensus on the treatment regime for both the acupuncture as well as the penetrating sham acupuncture group, we performed a Delphi consensus procedure [6]. Five experts from two major German acupuncture associations, the German Medical Acupuncture Association (Deutsche Ärztegesellschaft für Akupunktur; DÄGfA) Munich (n = 3), and the International Society for CM (Societas Medicinae Sinensis, SMS) (n = 2) Munich, participated in this consensus group and discussed the treatment regime with three experts on trial methodology and statistics in two onsite meetings and one written round. A consensus between the need for standardisation and individualisation was found in using a semi-standardised treatment in the acupuncture group.

The consensus procedure revealed the following result: Acupuncturists were requested to diagnose all their patients according to CM and to document the individual syndrome diagnosis for each patient from a given sample of possible diagnoses as the basis for an individualized, semi-standardised treatment in the acupuncture group [7].

Both the acupuncture and sham acupuncture treatments consisted of 12 sessions of 30 minutes’ duration administered over a period of 8 weeks (preferably 2 sessions a week for the first 4 weeks, followed by 1 session per week for the remaining 4 weeks). Patients in the rescue medication group did not receive acupuncture treatment during the first 8 weeks after randomisation; as of week 9, they received the acupuncture treatment described below.

Acupuncture treatment was semi-standardised (Table 1): All patients had to be treated at 4 obligatory basic acupuncture points: L. I. 4, L.I.11, L. I. 20 bilaterally and Ex-HN3 (Yintang). Furthermore, at least 3 of 8 optional basic acupuncture points had to be selected according to the principles of CM. In addition, patients had to be treated with at least 3 local and/or distant additional acupuncture points. Acupuncturists were allowed to use additional points including ear acupuncture points. Every acupuncture point used in this trial had to be documented after each session. Sterile and disposable single-use needles were used. Their length and type had to be documented. The treatment protocol aimed to produce the irradiating needling sensation (‘de qi’) if possible, and the needles were to be stimulated manually at least once in each session.

**Table 1:** Acupuncture points used in the ACUSAR trial

**Category of points**	**Points**	**Selection**
Basic acupuncture points (obligatory)	L.I. 4; L.I. 11; L.I. 20; EX-HN 3 YINTANG	Bilateral (except YINTANG), all together 7 points
Basic acupuncture points (optional)	EX-HN 8 BITONG GB 20 LIV 3 LU 7 ST 36 SP 6 SJ 17 BL 13	Uni- or bilateral, at least 3 points
Additional local acupuncture points (optional)	BL 2 GB 1, 14 EX-HN 5 TAIYANG SI 18, SJ 23 ST 2 Further points	At least 3 points (local and distant additional points) uni- or bilateral
Additional distant acupuncture points (optional)	LU 1, 5 ST 44 GB 41, 34, 37 LIV 2 LIV 5 KID 3, 7 BL 12, 20, 23, 26, 40 SP 9 REN 6, 17, 22, 20 SJ 5, 6 Further points	
Additional ear acupuncture (optional)	Allergy point Shenmen point Thymus point ACTH point Further points	

The location of points was performed on the basis of individual body size using measuring units equal to the transverse width of finger (TF), or ‘cun’. One cun is defined according to the traditional rules as the width of the interphalangeal joint of patient’s thumb.

Penetrating sham acupuncture treatment entailed superficially inserting fine needles (≤ 20 mm in length) at predefined, distant non-acupuncture points bilaterally (Table 2). These non-acupuncture points were not in the area of the face or head to avoid any possible local effects for SAR symptoms. The selection of at least 5 out of 7 points was left to the physician. Physicians were instructed to avoid manual stimulation of the needles and provocation of ‘de qi’ in the sham acupuncture group. All participating trial physicians received special training on how to apply this penetrating sham acupuncture; the training included a DVD that provided detailed instructions.

**Table 2:** ACUSAR trial: treatment in the acupuncture group

	**All sessions**	**Session 1**	**Session 6**	**Session 12**
	**n = 1.145**	**n = 97**	**n = 96**	**n = 93**
	**n (%)/mean±sd**	**n (%)/mean±sd**	**n (%)/mean±sd**	**n (%)/mean±sd**
**Number of needles/session**	10.0±1.6	10.0±1.6	10.0±1.5	9.9±1.5
**Duration of sessions (minutes)**	23.7±3.6	23.2±3.9	23.9±3.9	23.9±4.0
Length of needles used*				
≤ 15 mm	675 (59.0)	47 (48.5)	57 (59.4)	57 (61.3)
15 to 21 mm	521 (45.5)	49 (50.5)	43 (44.8)	40 (43.0)
20 to 30 mm	11 (1.0)	3 (3.1)	1 (1.0)	1 (1.1)
**Sham Acupuncture Points**				
- ‘Deltoideus’	1.130 (98.4)	97 (100.0)	94 (97.9)	91 (97.9)
- ‘Upper Arm’	1.079 (94.2)	93 (95.9)	90 (93.8)	87 (93.6)
- ‘Upper Thigh I’	1.037 (90.5)	87 (89.7)	87 (90.6)	84 (90.3)
- ‘Upper Thigh II’	1.011 (88.2)	89 (91.8)	84 (87.5)	81 (87.1)
- ‘Upper Thigh III’	1.019 (88.9)	88 (90.7)	85 (88.5)	82 (88.2)
- ‘Back I’	320 (27.9)	24 (24.7)	28 (29.2)	26 (28.0)
- ‘Back II’	186 (16.2)	12 (12.4)	17 (17.7)	15 (16.1)

* More than one answer possible.

Patients in all three trial arms were permitted to take up to two doses of cetirizine dihydrochloride/day. If SAR symptoms were not adequately controlled with cetirizine, participants could be treated with an oral corticosteroid. The use of other anti-allergy medication was prohibited. Patients were instructed to document the use of all anti-allergy medications precisely in their diaries. Patients were instructed not to use any of the following medications or treatments during the trial period in both years: Topical cromolyns (eye drops and nasal spray), topical antihistamines, topical steroids, leukotriene receptor antagonists, anti-cholinergic agents, α-adrenergic agonists, allergen immunotherapy, nasal ipratropium, decongestants and any form of complementary and alternative medicine (CAM) for SAR. In particular, no moxibustion or other additional complementary method was allowed.

All patients completed standardised questionnaires at baseline and after 8, 16, and 52 weeks. In addition, patients filled in diaries during the first 8 weeks and during weeks 14 to 16. Main outcome measures in the ACUSAR trial were the mean of the Rhinitis Quality of Life Questionnaire (RQLQ) overall score [8] and the mean of the Rescue Medication Score (RMS) [9] during the 7th and 8th week after randomisation. Rescue medication usage was scored daily using the RMS on a 4-point scale as follows: no rhinitis medication (0 points); oral antihistamines – 1 × cetirizine 10 mg/day or equivalent (1 point); oral antihistamines – 2 × cetirizine 10 mg/day (=20 mg) or equivalent, (2 points); any form of systemic steroids or other drug for SAR (3 points). Patient questionnaire included Visual Analogue Scales (VAS, 0–100 mm) for SAR overall symptom severity and for nasal, eye, pharyngeal and common symptoms, and the German version of the health-related quality of life instrument Short Form-36 (SF-36) [10,11]. Sample size was calculated on RQLQ data using nQuery Advisor, version 4.0, and assuming a power of 80% and a common SD of 1.1. More details on sample size calculation and statistical analyses have been published elsewhere [4].

## Results

**Figure 1:**
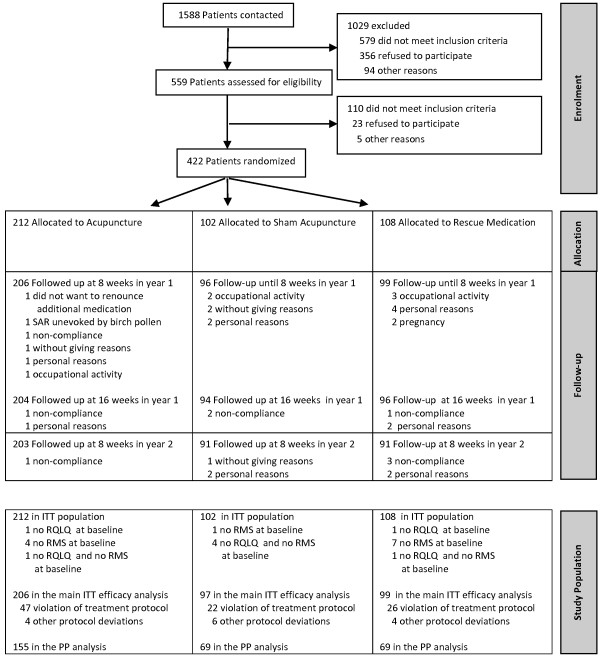
ACUSAR trial: patients’ flow chart.

Results of the ACUSAR trial have been published previously [4]. A total of 422 patients (Acu 212, Sham 102, RM 108; 60% female, 40% male; mean age 33 ± 8 years (SD) were included between March and May in both 2008 and 2009 (see Figure 1). 46 specialized physicians in 6 hospitals and 32 private outpatient clinics participated as trial centres. The majority of physicians were located in Bavaria (n = 21) and Berlin (n = 16) followed by North Rhine-Westphalia (n = 5), Brandenburg (n = 3) and Saxony (n = 1). Most centres recruited between 4 and 10 patients (n = 15), followed by centres that recruited more than 20 (n = 9), two and three patients (n = 8), and between 10 and 20 SAR patients (n = 6).

The characteristics of the 46 physicians providing trial intervention are summarised in Table 3. They had a median of 500 hours (range 140 to 2550 hours) of acupuncture training before participating in the trial, and 33 physicians (73%) had a ‘B - Diploma’. Sixteen (36%) trial physicians had taught acupuncture in accredited postgraduate courses. Physicians had used acupuncture in their respective practices for an average of 14 (1 to 30) years and had treated a median of 150 (15 to 5,000) patients and a median of 20 (0–550) SAR patients with acupuncture in the year preceding trial participation.

**Table 3:** ACUSAR trial: characteristics of participating trial physicians and assessment of intervention (n=46)

	**Mean ±sd; median (range) or n (%)**
Number of acupuncture sessions/centre	64.5; 48 (12-174)
Number of acupuncture and sham acupuncture sessions/centre	94.3; 72 (12-252)
Patients/Centre	6; 46 (27-69)
Age (years)	47±10; 46 (27-69)
Female	24 (52%)
Medical practice prior to study initiation (years)	20±11; 19 (1-46)
Postgraduate specialisation	31 (67%)
Postgraduate education	
- In acupuncture	31 (67%)
- In naturopathy	19 (42%)
- In homeopathy	2 (4%)
Acupuncture B diploma (at least 350 hours of training)	33 (73%)
Hours of acupuncture training	672±462; 500 (140-2550)
Teacher of acupuncture in accredited postgraduate courses	16 (35%)
Use of acupuncture prior to study initiation (years)	14±7; 15 (1-30)
Participation in earlier clinical trials	
- In general	34 (74%)
- On acupuncture	24 (52%)
- With randomisation	29 (63%)
- With sham or placebo acupuncture	21 (46%)
Membership in professional societies	
- In total	38 (83%)
- German Medical Acupuncture Association (DÄGfA)	16 (35%)
- International Society for Chinese Medicine (SMS)	16 (35%)
- German Association for Acupuncture and Neural Therapy (DGfAN)	4 (9%)
- In more than one acupuncture society	9 (20%)
- Others	10 (22%)
Patients treated with acupuncture by trial physicians in the year before the trial:	
- In total	420; 150 (15-5000)
- Patients with seasonal allergic rhinitis	58; 20 (0-550)
Therapies used in patients in everyday practice (percentages)	
- Acupuncture	41% (5-100%)
- Other Chinese Medicine therapies (e.g. Chinese herbal medicine)	28% (1-95%)
- Other complementary therapies	23% (1-90%)
- Conventional medicine	41% (2-100%)
Chinese diagnosis before treatment	
- Always	24 (52%)
- Frequently	20 (43%)
- Rarely	2 (4%)
- Never	0

Forty-four physicians (96%) stated that they frequently (43%) or always (52%) make a CM syndrome diagnosis before starting treatment.

A CM syndrome diagnosis was reported for all of the 422 patients who started the intervention (see Table 4). In 334 cases (79%), one (27.2%) or two CM syndrome diagnosis (51.4%) were reported. The most frequently reported primary syndrome diagnoses were “wind-cold invading the lung” and “wind-heat invading the lung” (both 37%), which includes the differentiation of typical symptoms for SAR such as nasal itching, sneezing, eye itching, followed by ‘lung and spleen deficiency’ (9%) and ‘liver heat’ (8%) as underlying syndromes. The most documented CM syndrome combinations were ‘wind-cold’ and ‘wind-heat invading the lung’ (24%) and ‘wind-heat invading the lung’ and ‘lung and spleen deficiency’ (15%).

**Table 4:** ACUSAR trial: Chinese medicine syndrome diagnoses (n=422)

**CM Diagnosis in ACUSAR patients**		**n (%)**
Patients with one CM diagnosis		117 (27.7)
Patients with two CM diagnoses		217 (51.4)
Patients with three CM diagnoses		88 (20.9)
**CM syndromes**	**TCM rank 1**	**TCM diagnoses**
	(n=422 patients)	(n=815 diagnoses)
	n (%)	n (%)
‘Wind cold’	156 (37.0)	221 (27.1)
‘Wind heat’	155 (36.7)	238 (29.2)
‘Liver heat’	33 (7.8)	105 (12.9)
‘Chronic heat, yin deficiency’	16 (3.8)	37 (4.5)
‘Chronic heat in the lung’	21 (5.0)	55 (6.7)
‘Lung and spleen qi deficiency’	36 (8.5)	132 (16.2)
‘Yang deficiency in kidneys’	5 (1.2)	27 (3.3)
**Combined CM syndroms**	**TCM rank 1 and 2**	
‘Wind cold’ & ‘wind heat in the lung’	74 (24.3)	
‘Wind heat’ & ‘lung and spleen qi’	38 (12.5)	
‘Wind cold’ & ‘lung and spleen qi’	47 (15.4)	
‘Wind heat’ & ‘liver heat’	45 (14.8)	
Others	101 (33.1)	

Patients in the acupuncture group were treated in a total of 2.455 sessions. According to the protocol, all of the patients were treated at obligatory and optional basic acupuncture points. On average, 15.7 ± 2.5 (mean and standard deviation) needles were used per session and the mean duration of each acupuncture session was 24.4 ± 4.4 minutes. The number of needles per session remained stable over the course of treatment. In almost all patients (>93%), the ‘de qi’ sensation could be elicited, and in most cases (>80%) manual stimulation was performed once. Length of needles used was in most cases a combination of 21 to 30 mm (84%) and ≤ 20 mm (73%). All basic obligatory points were used in 97% of cases, L.I. 4 and L.I. 11 were used in 100% of cases. Basic optional acupuncture points used most frequently were GB 20, LIV 3, ST 36, LU 7 and SP 6. For the optional basic acupuncture points, more than 3 acupuncture needles were used in 95% of cases. Additional local and distant acupuncture points were used in 63% and 81% of cases. In 39% of cases, additional ear acupuncture points were used (see Table 5).

**Table 5:** ACUSAR trial: treatment in the acupuncture group

	**All sessions**	**Session 1**	**Session 6**	**Session 12**
	**n = 2.455**	**n = 210**	**n = 205**	**n = 201**
	**n (%)/mean±sd**	**n (%)/mean±sd**	**n (%)/mean±sd**	**n (%)/mean ±sd**
**Number of needles/session**	15.7±2.5	15.7±2.5	15.8±2.5	15.7±2.7
**Duration of session (min)**	24.4±4.4	24.3±4.5	24.5±4.6	24.6±4.7
Length of needles used*				
≤ 20 mm	1.784 (72.7)	156 (72.9)	151 (73.7)	147 (73.1)
21 to 30 mm	2.053 (83.6)	171 (81.4)	173 (84.4)	162 (81.0)
31 to 40 mm	846 (34.5)	70 (33.3)	70 (34.2)	75 (37.5)
≥ 40 mm	103 (4.2)	10 (4.8)	9 (4.4)	10 (5.0)
**Manual stimulation**				
- None	259 (10.5)	16 (7.6)	25 (12.2)	32 (16.0)
- Once	1.966 (80.1)	172 (81.9)	162 (79.0)	152 (76.0)
- More than once	230 (9.4)	22 (10.5)	18 (8.8)	16 (8.0)
**De qi**				
- Easy to elicit	2.276 (92.9)	186 (88.6)	191 (93.2)	189 (94.5)
- Difficult to elicit	169 (6.9)	23 (11.0)	14 (6.9)	10 (5.0)
- Could not be elicited	10 (0.4)	1 (0.5)	0	1 (0.5)
**Basic points (obligatory and optional)** Needles/session	12.3±1.9	12.3±1.9	12.3±1.9	12.3±1.9
**Basic obligatory points**				
- L.I.4 (right & left)	2.455 (100)	210 (100)	205 (100)	200 (100)
- L.I.11(right & left)	2.455 (100)	210 (100)	205 (100)	200 (100)
- L.I.20 (right & left)	2.412 (98.2)	206 (98.1)	201 (98.1)	198 (98.5)
- Ex-HN3 (Yintang)	2.432 (99.1)	208 (99.1)	203 (99.0)	199 (99.0)
- 7 Points used	2.378 (96.9)	205 (97.6)	198 (96.6)	194 (97.0)
**Basic optional points**				
- GB20 (right & left)	1.567 (63.8)	131 (62.4)	131 (63.9)	129 (64.5)
- LIV3 (right & left)	1.253 (51.0)	138 (65.7)	136 (66.3)	133 (66.2)
- LU7 (right & left)	1.201 (48.9)	101 (48.1)	101 (49.3)	98 (49.0)
- ST36 (right & left)	1.478 (60.2)	126 (60.0)	123 (60.0)	121 (60.5)
- SP6 (right & left)	1.028 (41.9)	90 (42.9)	86 (42.0)	63 (41.5)
- SJ17 (right & left)	48 (2.0)	4 (1.9)	4 (2.0)	4 (2.0)
- BL13 (right & left)	204 (8.3)	17 (8.1)	17 (8.3)	17 (8.5)
- Ex-HN8 (right & left)	474 (19.3)	40 (19.1)	40 (19.5)	40 (19.4)
- Points used >=3	2.343 (95.4)	199 (94.8)	196 (95.6)	191 (95.5)
**Additional optional points** Needles/session	3.5 ± 1.9	3.5 ± 1.9	3.5 ± 1.9	3.5 ± 1.9
- Local (sessions)	1.550 (63.1)	131 (62.4)	129 (62.9)	128 (63.7)
- Distant (sessions)	1.991 (81.1)	169 (80.5)	167 (81.5)	162 (81.0)
- Points used>=2	2.292 (94.5)	198 (94.3)	194 (94.6)	190 (94.5)
**Additional ear acupuncture**	967 (39.4)	82 (39.1)	81 (39.5)	78 (39.0)

*More than one answer possible.

Patients in the sham acupuncture group were treated in a total of 1.145 sessions due to the 2:1:1 randomisation (see Table 6). On average, 10.0 ± 1.6 (mean and standard deviation) needles were used per session and the mean duration of each acupuncture session was 23.7 ± 3.6 minutes. Length of needles used solely or in combination was ≤ 15 mm (59%) and 15 to 21 mm (46%). The most frequently used sham acupuncture points were ‘Deltoideus’ , ‘Upper Arm’ , ‘Upper Thigh I’ , ‘Upper Thigh II’ , and ‘Upper Thigh III’.

**Table 6:** ACUSAR trial: treatment in the sham acupuncture group

	**All sessions**	**Session 1**	**Session 6**	**Session 12**
	**n = 1.145**	**n = 97**	**n = 96**	**n = 93**
	**n (%)/mean±sd**	**n (%)/mean±sd**	**n (%)/mean±sd**	**n (%)/mean±sd**
**Number of needles/session**	10.0±1.6	10.0±1.6	10.0±1.5	9.9±1.5
**Duration of sessions (minutes)**	23.7±3.6	23.2±3.9	23.9±3.9	23.9±4.0
Length of needles used*				
≤ 15 mm	675 (59.0)	47 (48.5)	57 (59.4)	57 (61.3)
15 to 21 mm	521 (45.5)	49 (50.5)	43 (44.8)	40 (43.0)
20 to 30 mm	11 (1.0)	3 (3.1)	1 (1.0)	1 (1.1)
**Sham acupuncture points**				
- ‘Deltoideus’	1.130 (98.4)	97 (100.0)	94 (97.9)	91 (97.9)
- ‘Upper Arm’	1.079 (94.2)	93 (95.9)	90 (93.8)	87 (93.6)
- ‘Upper Thigh I’	1.037 (90.5)	87 (89.7)	87 (90.6)	84 (90.3)
- ‘Upper Thigh II’	1.011 (88.2)	89 (91.8)	84 (87.5)	81 (87.1)
- ‘Upper Thigh III’	1.019 (88.9)	88 (90.7)	85 (88.5)	82 (88.2)
- ‘Back I’	320 (27.9)	24 (24.7)	28 (29.2)	26 (28.0)
- ‘Back II’	186 (16.2)	12 (12.4)	17 (17.7)	15 (16.1)

*More than one answer possible.

In the first 8 weeks, the proportion of patients who used 10 mg and/or 20 mg cetirizine was 71% in the acupuncture group, 76% in the sham acupuncture group, and 83% in the RM group. Oral steroids were used by 3 patients, one in each group. Altogether 27 patients (16 acupuncture, 4 sham acupuncture and 7 RM) took anti-allergy medication (mostly topical steroids and cromoglicic acid, intake less than 12 days) not permitted in the trial.

## Discussion

Our analyses demonstrate that the physicians participating in the ACUSAR trial were a heterogeneous group whose overall qualifications included extensive acupuncture training and long-term acupuncture experience that clearly exceeded not only the qualification required for trial participation but also the qualification of 200 h acupuncture training required for the German statutory reimbursement of acupuncture treatment [12]. In addition, our data indicate that the consensus-based treatment protocol used for acupuncture and sham acupuncture in this trial was a feasible and successful approach. Finally, in the ACUSAR trial, acupuncture plus RM led to improvements in disease-specific quality of life and reduction of antihistamine intake after 8 weeks of treatment compared to sham acupuncture plus RM and to RM alone in SAR patients [4].

High quality randomised trials of acupuncture compared to usual care or sham acupuncture are urgently needed to evaluate both the effectiveness and efficacy of this widely used CM intervention for various indications including SAR. However, defining interventions in randomised controlled acupuncture trials represents a challenging task. From a strictly scientific point of view, standardised interventions are needed so that the findings of a trial can be reproduced independently. To date, three trials [13-15] comparing acupuncture and sham acupuncture interventions in SAR have been published. In contrast to the negative trials of Williamson and Magnusson, in our trial and in the other positive trial from Xue [15], a semi-standardised and therefore a more individualised acupuncture intervention based on a CM syndrome diagnosis was used.

Acupuncture in Germany and elsewhere is applied in a highly variable manner. Treatment is often individualised, as many physicians and acupuncturists believe that this results in the greatest effectiveness [16]. Because of this, acupuncture trials that use strictly standardised interventions might neither represent real treatment conditions nor an adequate foundation for guiding health care policy decisions on acupuncture treatment. In the ACUSAR trial, as well as in our ART trials published previously [17-20], we opted for a compromise that would ensure a fundamental degree of consistency while at the same time allowing some level of individualisation with regard to point selection. The pre-published treatment protocol was developed as part of a consensus process involving leading experts from two German medical acupuncture societies. The approach to medical acupuncture in Germany is, in general, based more on the theories of CM. We think that our pragmatic intervention approach is consistent with the theory of CM and may yield better results in patients with SAR than a strictly standardized acupuncture. However, we cannot rule out that our trial may have resulted in different outcomes if we had used a different acupuncture intervention in the acupuncture and sham acupuncture groups.

The authors of another large trial that included 238 persistent allergic rhinitis patients and compared acupuncture with both sham-acupuncture and waiting list published their results in 2013 [21]. Similar to our trial, patients were treated with 12 acupuncture sessions including a penetrating procedure for the sham acupuncture. In contrast to the ACUSAR trial, the acupuncture intervention was strictly standardised using 10 defined points including bilateral L.I.4, L.I.20, ST2, ST36 and unilateral EX-HN3 (Yin Tang) and GV23. This trial yielded positive results for the main outcome parameter, the Total Nasal Symptom Score (TNSS) in favour of acupuncture versus sham-acupuncture and usual care. However, the results of this trial, (unlike those in the ACUSAR trial), were inconsistent, because the secondary outcome parameter e.g., Total Non-Nasal Symptom Score (TNNSS) and the Rhinitis Quality of Life (RQLQ) didn’t show positive results for acupuncture compared to sham acupuncture.

It is noteworthy that in our trial the number of acupuncture points in the acupuncture group was on average 15.7 ± 2.5 and therefore different compared to the number of acupuncture points in the sham acupuncture group, which averaged 10.0 ± 1.6. Further, because the selection of possible sham acupuncture points was smaller compared to those points in the acupuncture treatment scheme, the sham acupuncture scheme was even more standardised than treatment in the acupuncture group. We are aware that these differences, particularly the higher number of needles in the acupuncture group, could explain the better clinical effect in SAR patients. Although difficult in a semi-standardised treatment, for future trials it might be more appropriate to ensure that acupuncture and sham acupuncture groups are each treated by a similar number of needles.

Even more difficult than defining the acupuncture intervention itself is the choice of an appropriate sham control. The German Research Foundation (DFG) requested that our trial include a ‘sham’ or ‘placebo’ condition to investigate whether the effects of acupuncture are specific. However, the concepts of ‘placebo’ and ‘specific effects’ are unclear in relation to acupuncture [22]. Although it is widely accepted in CM that it is important to correctly locate points, it should be noted that this theory has yet to be proved. Indeed, other aspects of acupuncture treatment, such as skin penetration, depth of needling, manipulation of needles, etc., may also be relevant effect modifiers. In the absence of an inert and indistinguishable placebo, a wide variety of sham interventions have been used in acupuncture trials. Based on a systematic review of such interventions [23], as well as on our consensus procedure with acupuncture experts, we decided to use ‘sham’ acupuncture [24] as a sham control. It differed from the ‘full’ or ‘true’ acupuncture intervention with regard to point location, needling depth, and the avoidance of ‘de qi’ and manual needle stimulation. Similar interventions have been used in a variety of previously published trials including all ART trials [18,19,23]. We think a pragmatic comparison between an acupuncture treatment *lege artis* and a form that is clearly not following standard acupuncture theory and practice appears to be an acceptable compromise.

In contrast to the other acupuncture trials, physicians in our study were asked to provide a CM syndrome diagnosis for all patients. This was a basis for the semi-standardised, individualized intervention in the acupuncture group. The most frequent syndrome diagnoses in SAR patients included in the trial were ‘wind-cold invading the lung’ and ‘wind-heat invading the lung’. This distribution of syndromes in SAR patients corresponds with the statements of available textbooks on the subject [7,25]. Several acupuncture points are possible for the treatment of these syndromes. Textbooks recommend points like inter alia L.I.4, L.I. 11, LU 7, GB 20, SP 6, ST 36, LIV 3 which were respectively obligatory or the most chosen optional points in ACUSAR. Only the Xue [15] trial included an obligatory syndrome differentiation and it is interesting to discover that Xue used a slightly modified syndrome differentiation more focused on the CM organ system rather than on the physiopathology of the CM syndrome.

## Conclusion

In conclusion, ACUSAR trial interventions were provided by well educated and experienced acupuncturists and contained more needles in the acupuncture group compared to the sham acupuncture group. For future trials, it might be more appropriate to ensure that acupuncture and sham acupuncture groups are each treated by a similar number of needles. Leaving this important point aside, we think that the trial intervention protocol for acupuncture in the ACUSAR trial represented an acceptable and feasible compromise between an acupuncture treatment following the rules of CM and the daily practice in Germany and the standardisation need in clinical research.

## Competing interests

None of the authors declare any financial or non-financial competing interests.

## Authors’ contributions

BB conceived the trial together with CW, KL and SW. BB drafted the manuscript together with MO; BB was responsible for trial coordination. JH, DI, MW, FP, BB, and MO participated in the design of the acupuncture and sham acupuncture treatment and/or took part as trial physicians. BN was involved for special questions on allergy and rescue medication. SB was responsible for data management. CH participated in data processing for the manuscript. All authors read and approved the final manuscript.

## Pre-publication history

The pre-publication history for this paper can be accessed here:

http://www.biomedcentral.com/1472-6882/14/128/prepub
